# Retinal blood vessel‐origin yes‐associated protein (YAP) governs astrocytic maturation via leukaemia inhibitory factor (LIF)

**DOI:** 10.1111/cpr.12757

**Published:** 2020-01-08

**Authors:** Li‐Qian‐Yu Ai, Rong‐Di Yuan, Xi Chen, Yun‐Jia Liu, Wen‐Yi Liu, Jing‐Yi Zhu, Zhou Zhang, Jun Yan, Chun‐Lin Chen, Sen Lin, Jian Ye

**Affiliations:** ^1^ Department of Ophthalmology Research Institute of Surgery & Daping Hospital Army Medical Center of PLA Army Medical University Chongqing China; ^2^ Department of Ophthalmology XinQiao Hospital Army Medical University Chongqing China; ^3^ Research Institute of Surgery & Daping Hospital Army Medical Center of PLA Army Medical University Chongqing China

**Keywords:** astrocyte premature, leukaemia inhibitory factor, retinal vessel development, yes‐associated protein

## Abstract

**Objectives:**

To testify that endothelial cells (ECs) induce astrocyte maturation by leukaemia inhibitory factor (LIF) secretion.

**Materials and Methods:**

In vivo experiments, mice bearing floxed alleles of YAP were crossed with mice expressing a Cre recombinase driven by the endothelial *Tek* promoter (*Tek*‐Cre) to finally obtain the following three genotypes: *YAP* ^f/f^, *Tek*‐Cre; *YAP* ^f/w^, *Tek*‐Cre; and *YAP* ^f/f^. Retinal vascularization and astrocyte network were evaluated by whole‐mount fluorescence and Western blotting. In vitro, experiments were performed in an astrocyte and human microvascular endothelial cell (HMEC‐1) coculture model to analyse the mechanisms underlying the effect of endothelial YAP on astrocytes.

**Results:**

In vivo, YAP^f/f^;Tek‐Cre mice showed delayed angiogenesis, sparse vessels and decreased glial fibrillary acidic protein (GFAP)+ astrocytes but aberrant growth of endothelial networks and immature astrocytes (platelet‐derived growth factor A, PDGFRA+ astrocytes) overgrowth. In vitro, Yap deletion attenuated the LIF release that delayed the maturation of retinal astrocyte which was consistent with the results of HMEC‐1—astrocyte coculture. The effect of YAP overexpression on LIF‐LIFR axis in HMEC‐1 interferes the GFAP expression of astrocyte. In contrast, LIF protein rescues the astrocytic GFAP expression when EC YAP was inhibited by siRNAs.

**Conclusions:**

We show that EC yes‐associated protein (YAP) is not only a critical coactivator of Hippo signalling in retinal vessel development but also plays an essential role in retinal astrocyte maturation by regulating LIF production.

## 1 INTRODUCTION

The dynamic interactions between astrogliogenesis and blood vessel growth in physiological and pathological conditions have long been of interest. Mechanisms for colonization must result in specific cell‐cell interactions that are critical for glial function.^1^ The peak period of gliogenesis coincides with the rapid growth of blood vessel surfaces in rodent brains.^2^, ^3^, ^4^ In the retina, vascular patterning is essentially dependent on astrocytes in physiological conditions, and disruption of this process severely impairs vascular growth.^5^, ^6^ Astrocytes contribute to the breakdown of the blood‐brain barrier and neovascularization in retinal pathological conditions such as oxygen‐induced retinopathy (OIR) and blood‐retinal barrier (BRB) breakdown.^7^

Conversely, the physiological and pathological conditions of vessels contribute to astrocyte differentiation, which is critically important in glial‐neurovascular unit development and pathology.^8^ Delayed vascularization leads to abnormal astrocyte behaviour and endothelial cell (EC) networks in the mouse retina.^8^, ^9^ Recent studies have demonstrated that the transcriptional coactivator yes‐associated protein (YAP) affects EC development and angiogenesis. YAP depletion inhibits endothelial cell tube formation and leads to stunted sprouting with local aggregation as well EC scarcity, branching irregularities and junction defects.^10^, ^11^, ^12^ During postnatal development of the mouse retina, YAP has been shown to regulate vascular branching and density by promoting the transcription of angiopoietin‐2.^13^, ^14^ Loss of YAP and its paralog WW domain‐containing transcription regulator 1 (TAZ) decreases VE‐cadherin turnover and the frequency of junction‐associated intermediate lamellipodia.^11^ YAP is also recognized as a biosensor in the stimulation of EC growth by αvβ3 integrin activation^15^ and regulated by EC and other types of cell‐cell contacts.^13^, ^16^, ^17^, ^18^

Although the essential roles of YAP in ECs vascularization have been revealed, the mechanisms underlying retinal vessel and astrocyte contact and retinal astrocyte differentiation remain unclear. Substances secreted from ECs, such as leukaemia inhibitory factor (LIF),^19^ which is predominantly expressed in the developing endothelium,^20^ have been shown to change astrocyte characteristics.^19^, ^20^ LIF receptor (LIFR) expression in the surrounding astrocytes has been shown to be involved in retinal vascularization.^20^ The retina is an excellent model to study the role of cell type‐specific contributions to the process of blood vessel and neuronal growth. In this study, using *Tek*‐Cre; *YAP* ^f/f^ mice, we showed that retinal vascularization and astrocyte maturation were retarded depending on endothelial LIF‐astrocytic LIFR behaviour. Therefore, these mechanisms of the interactions between retinal vessels and astrocytes provide potential therapeutic targets for retinal neovascularization.

## 2 MATERIALS AND METHODS

### 2.1 Reagents

YAP (ab205270), GFAP (ab7260), CD31 (ab28364), PDGFR alpha (ab203491), GAPDH (ab8245), LIF (ab113262),LIFR (ab101228), Claudin (ab15106), aldolase C (ab87122), Vimentin (ab8978), Vimentin (ab92547), and Goat Anti‐Rabbit IgG H&L (Alexa Fluor® 488) (ab150077), Goat Anti‐Mouse IgG H&L (Alexa Fluor® 488) (ab150113), Goat Anti‐Rabbit IgG H&L (Alexa Fluor® 594)(ab150080), Goat Anti‐Mouse IgG H&L (Alexa Fluor® 594) (ab150116), Goat Anti‐Mouse IgG H&L (Alexa fluor® 647) (ab150115), Goat Anti‐Rabbit IgG H&L (Alexa Fluor® 647)(ab150079) and EGF protein (ab126695) were purchased from Abcam (Cambridge, UK). GFAP (MAB360) was purchased from Millipore (Massachusetts, USA). ZO1 (617300) was purchased from NOVEX by life technologies (Massachusetts, USA). YAP1 (3A79) was purchased from Proteintech group (Chicago, USA). Dil stain (1,1'‐Dioctadecyl‐3,3,3',3'‐Tetramethylindocarbocyanine Perchlorate (D282)) was purchased from Thermo Fisher (Massachusetts, USA). Transfected constructs LV‐efla‐yap1‐EGFP‐WPRE and LV‐efla‐EGFP‐WPRE were purchased from Brain VTA (WuHan, China). Sequence‐based reagent: siRNA YAP and siRNA LIFR were purchased from RIBOBIO (GuangZhou, China). Protein reagent: LIF protein and PDL (poly‐d‐lysine) were purchased from Sigma‐Aldrich (St Louis, USA). Agonist reagent: Y27632 dihydrochloride was purchased from MedChemExpress (New Jersey, USA). Isolectin GS‐IB4 Alexa Fluor™ 488 (121411) and 594 (121413) were purchased from Invitrogen (Massachusetts, USA). MCDB131 Medium, DMEM (Dulbecco's Modified Eagle Medium), DMEM/DF12, FBS (foetal bovine serum), streptomycin (0.1 mg/mL) and penicillin (100 U/mL) were purchased from Gibco (Massachusetts, USA).

### 2.2 Animals: *YAP* ^f/f^;*Tek*‐Cre mice

For loss‐of‐function experiments, the following mouse strains were used: *YAP* ^f/f^ mice: B6.129P2(Cg)‐YAP1 ^tm1.Dupa^/J (J032192) bred with *Tek*‐iCre mice: B6.Cg‐Tg (Tek cre)12Flv/NJU (J004128). Mice bearing floxed alleles of YAP were crossed with mice expressing a Cre recombinase driven by the endothelial *Tek* promoter (*Tek*‐Cre) to finally obtain the following three genotypes: *YAP* ^f/f^, *Tek*‐Cre; *YAP* ^f/w^ and *Tek*‐Cre; and *YAP* ^f/f^. In all experiments, control animals (*YAP* ^f/f^ mice) were littermates not expressing Cre. Male and female mice were used for the analysis. Mouse genotypes were determined by PCR analysis of genomic DNA that was isolated from mouse tails using the 2 and 3 primer sets targeted to the adjacent Cre and *YAP* genes, respectively. All the animals analysed in this study were from at least three separate litters. This study was carried out in accordance with the recommendations of ARRIVEA (Animal Research: Reporting In Vivo Experiments Guidelines) guidelines in accordance with the National Institutes of Health guide for care and use of Laboratory animals. The protocol was approved by the Animal Ethics Committee of AMU.

### 2.3 Cell culture

#### 2.3.1 HMEC‐1

HMEC‐1 (Human Microvascular Endothelial Cell, CRL‐3243™) from the American Type Culture Collection (ATCC) was cultured in MCDB131 containing 10% FBS, 10 ng/mL epidermal growth factor (EGF), 1 µg/mL hydrocortisone, and 10 mmol/L glutamine.

#### 2.3.2 Astrocytes

Cultured astrocytes were prepared from the cerebral cortices of newborn C57BL/6 mice (1 day old). First, the culture plates were incubated in poly‐D‐lysine (PDL) overnight and washed with DF12 medium for preparation. Then, the meninges were removed, and the cortex was washed with PBS and cut into small cubes (1 mm^3^). Then, 0.05% tryptase (at a volume 30‐50 times more than the total amount of tissue mass) was added to the cubes, which were then disrupted by moderate vortexing. The digestion was suspended by the addition of DMEM/F12 (containing 10% FBS). After centrifugation at 1000 r/min for 5 minutes, the cell pellets were resuspended in DMEM/F12 (containing 10% FBS), and the medium was changed every 3 days until astrocyte growth was observed. The incubator was maintained at 37°C in an atmosphere containing 5% CO_2_, air and 90% humidity.

### 2.4 Fluorescence immunostaining in whole‐mount retinas

#### 2.4.1 Mice retinal perfusion

After injection of anaesthetics, the abdomen area of the mice was cut open with the heart exposed. A 1 mL insulin syringe was filled with saline, acupunctured into the left ventricle (the apex of the heart) and injected slowly after cutting open the auricula dextra. Perfusion was stopped until the fluid had become colourless and transparent from the right atrium, after 5‐10 mL of saline. Then, the left ventricle was injected with another syringe filled with 4% paraformaldehyde of the same volume. (If Dil stain is required, an additional step of the dye injection was added before the 4% paraformaldehyde, and the method of perfusion was the same as before).

#### 2.4.2 Whole‐mount retina preparation

The eyes were enucleated by curved forceps and immediately transferred to a tissue culture plate filled with 4% paraformaldehyde (PFA). The eye tissue samples were fixed for 30 minutes at room temperature and then placed in a pool of PBS under a dissecting microscope for retina isolation. Here, the cornea and optic nerve were pinched with forceps; when the cornea was secured, the hold on the optic nerve was released, and a surgical blade was obtained with the free hand to make a radial incision on the cornea. Starting at the incision, the sclera was carefully peeled away towards the optic nerve with forceps. The cornea, sclera, optic nerve, retina pigment epithelium and lens were removed and discarded, leaving only the retina. Finally, the retinal cup was cleaned, removing all debris, loose vessels and hyaloid vessels, and intact retinas were dissected into four parts by forceps.

#### 2.4.3 Fluorescence immunostaining

Retinas were blocked and permeabilized in goat serum that contained 0.5% Triton X‐100 overnight at 4°C. Then, retinas were incubated with different antibodies (targeting GFAP or/and PDGFRA) for 2 days at 4°C and fluorescence‐conjugated secondary antibodies overnight at 4°C. Finally, retinas were incubated with IB4 for 1 day at room temperature. Retinas were washed with PBS between incubations and carefully mounted on microscope slides in mounting medium. Immunostained retinas were examined by confocal laser scanning microscopy (Leica, Germany) and were scanned by z‐stack from the top layer to the deep layer. Areas, vessels length, junctions, end points and tip sprout numbers of retinal vascular and astrocyte networks were quantified using AngioTool (University of Warwick, UK). Eight nonoverlapping and randomly selected microscopic fields per retina were imaged by confocal scanning laser microscopy to assess the formation and structure of ECs and astrocytes.

#### 2.4.4 Frozen section of retina

Perfusion, fixation and other methods are the same as above, with dehydration of 20% sucrose. OCT was embedded at −80° overnight and was sectioned on the second day at −20° with a thickness of 8‐10 microns. The staining and confocal steps were the same as above.

### 2.5 Reagents, treatment and cell transfection

For YAP gain‐of‐function experiments, HMEC‐1 cells were transfected with LV‐efla‐yap1‐EGFP‐WPRE at different concentrations (5, 10, 20 µm) for 48 hours according to the specifications. LV‐efla‐EGFP‐WPRE was used as a control, and the blank control (NC) group received no additional transfection. For LIF overexpression experiments, HMEC‐1 cells were cultured with LIF protein (10 µmol/L) for 48 hours, and the NC group was cultured with no LIF protein. For YAP or LIFR knockdown experiments, HMEC‐1 cells or astrocytes were transfected using SMARTpool: briefly, subconfluent (30%‐50%) HMEC‐1 cells or astrocytes were transfected with 50 nmol/L siRNA using transfection reagent following the protocol from the manufacturer, and experiments were routinely performed 48 hours after transfection.

### 2.6 Cell and tissue Western blot

HMEC‐1 cells and astrocytes were inoculated in 6‐well plates. After successful adherence and growth of astrocytes (approximately 3‐5 days) and HMEC‐1 cells (overnight), the cells were transfected with their own reagents (LV, siRNA or LIF protein) for 48 hours. The cells or culture medium were then collected. Astrocytes in the coculture system were transfected with their own siRNA for 48 hours and then cultured in the transfected medium from HMEC‐1 cells until they were used for the experiments.

The retina tissues were prepared following the whole‐mount retina preparation protocol without 4% PFA.

Following treatment, cells or tissues were washed thoroughly in cold PBS (0.1 mol/L, pH 7.4) and incubated in 100 μL cold lysis buffer (Thermo Fisher Scientific, Waltham, UK) for 30 minutes. Then, the cells or tissues were completely disrupted by ultrasound for 3 minutes. The supernatant containing total proteins was collected after centrifugation at 12 000 *g* for 15 minutes at 4°C. Protein from each group was tested using a BCA protein quantitation kit, diluted with loading buffer (Beyotime Biotechnology, Nantong, China), boiled and stored at −20°C.

Equal amounts (25 μg) of proteins from each group were electrophoresed on 10% or 12% SDS‐PAGE gels and then transferred to polyvinyl difluoride (PVDF) membranes (Roche, Basel, Switzerland). After the membranes were blocked with 7% milk in Tris‐buffered saline (TBST, pH 7.4) at room temperature for 3 hours, they were incubated with different primary antibodies (following the specification) at 4°C overnight. After the membranes were washed in TBST 3 times for 15 minutes, they were incubated with different secondary antibodies (following the specification) at room temperature for 2 hours. Immunoreactive bands were developed by Immobilon Western Chemiluminescent HRP Substrate (Vilber‐Lourmat, France) according to the manufacturer's instructions. All bands were quantified using Photoshop software (USA), and band densities were normalized with respect to the glyceraldehyde‐3‐phosphate dehydrogenase (GAPDH) values.

### 2.7 Immunocytochemistry

HMEC‐1 cells and astrocytes were inoculated in 6‐well plates and 24‐well plates. After successful adherence and growth of astrocytes (approximately 3‐5 days) and HMEC‐1 cells (overnight), the cells were transfected with their respective reagents (LV, siRNA or LIF protein) for 48 hours, and then, slides were put into 6‐well plates with HMEC‐1 cells to maintain the coculture conditions until the experiments were completed. For labelling of GFAP, KI67 or IB4, cells were fixed with 4% PFA for 30 minutes and washed in PBS three times. Then, cells were incubated in a blocking solution containing 0.05% Triton X‐100 in goat serum for 1 hour at 37°C and stained with each primary antibody or IB4 overnight at 4°C following the specified dilution. After incubation, cells were washed with PBS again, and nuclei were stained with 4', 6‐diamidino‐2‐phenylindole (DAPI) reagent (ZSGB‐Bio, BEIJING, CHINA). The images were acquired using a confocal laser scanning microscope (Leica, Germany), and the fluorescence intensity and cell number were counted by Image‐Pro. Each experiment was repeated at least 3 times.

### 2.8 AngioTool Soft

AngioTool was available as a free download on https://ccrod.cancer.gov/confluence/display/ROB2/Home. AngioTool was started, parameter was set to “vessel diameter and intensity” (to ensure the consistency of each experiment), and analysis was conducted on the confocal images. The resulting data include the following: vessel percentage, vessel length, end points and junctions.

### 2.9 Statistics

All of the statistical tests were performed using SPSS 18.0 (IBM, Chicago, USA), and statistical graph was using Sigma plot 12.5 (SYSTAT, USA). All experiments were performed at least three times. The significance of the differences among groups was analysed by one‐way ANOVA, and comparisons for two groups of data were done by t test. Results were presented as the mean ± SEM. *P*‐value of less than .05 was considered to indicate statistical significance.

## 3 RESULTS

### 3.1 YAP is critical for retinal vessel development

**Figure 1:**
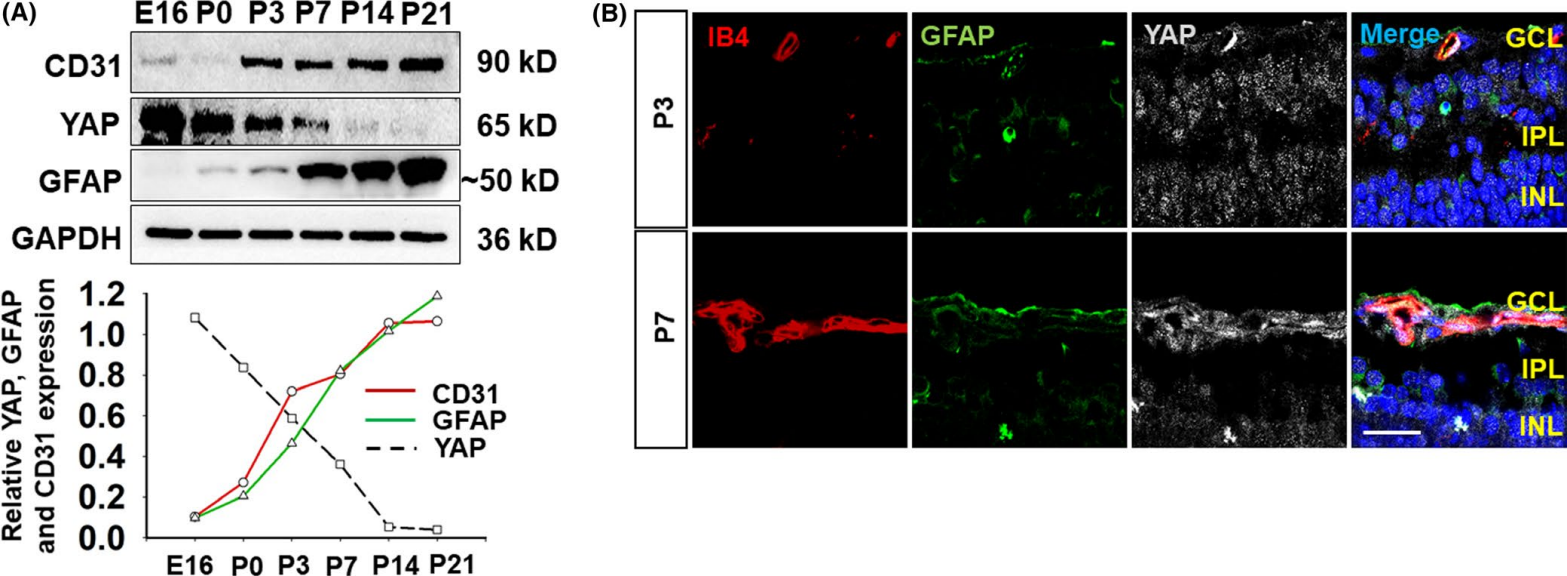
Yes‐associated protein is critical for retinal vessel development. A, CD31, GFAP and YAP protein expression in early retinal growth from E16 to P21 tested by Western blot. The chart shows the trend of different protein expression, CD31 in red line, GFAP in green line and YAP in black Dotted line. B, Colocalization of YAP (in grey) with markers of blood vessels (in red) and astrocytes (in green) in the GCL at P3 and P7 was shown by immunofluorescence analysis of tissue sections, bar = 20 μm. N = 3 in (A, B)

According to previous studies, astrocytes and blood vessels depend on each other to modulate retina development.^21^ We used Western blot to confirm that platelet endothelial cell adhesion molecule‐1 (PECAM‐1/CD31) and glial fibrillary acidic protein (GFAP) expression increased dramatically from embryonic day 16 (E16) to postnatal day 21 (P21) (Figure 1A). Fluorescence immunostaining with isolectin B4 (IB4) and anti‐GFAP showed accordant localization of endothelium and astrocytes, respectively, from P3 to P21 (Figure S1A,B). YAP, a transcriptional coactivator in Hippo signalling, is an important regulator of angiogenesis,^11^ as well as retinal vascular development. However, the effect of YAP on the relationship between astrocyte growth and blood vessels needs to be elucidated. For the retinal development procedure, we examined the expression pattern of YAP from E16 to P21 and observed an obviously decreasing tendency (Figure 1A). YAP predominately affects early retinal growth, especially from E16 to P7, which is also a critical period for the development of blood vessel sprouting and astrocyte maturation.^21^ Here, we observed the localization of YAP in IB4‐positive retinal vessels and in retinal progenitor cells at P3 (Figure 1B), which is consistent with a previous report.^22^ In addition, specific localization of YAP was mainly found in IB4‐positive blood vessels at P7 (Figure 1B), with very little staining in retinal progenitors.

Herein, we identified the specific localization of YAP in the developing retina. Our results suggest that YAP might be closely related to changes in astrocytes and blood vessels. However, the role of YAP in the relationship between retinal astrocytes and blood vessels required further exploration.

### 3.2 *YAP* ^f/f^;*Tek*‐Cre mice exhibit increased astrocyte irregularity in early retinal growth

**Figure 2:**
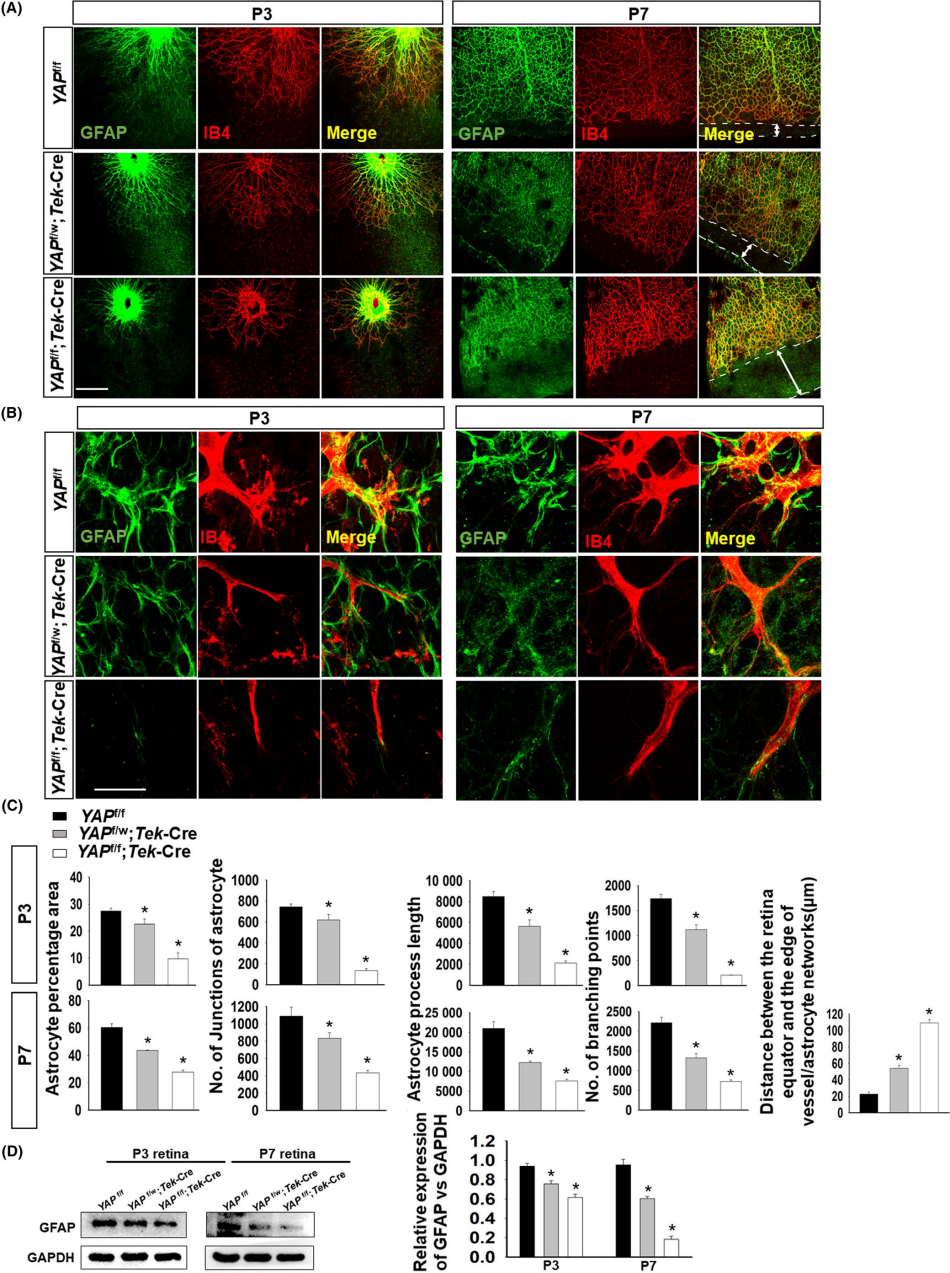
*YAP* ^f/f^;*Tek*‐Cre mice show increased irregularity of astrocytes in early retinal growth. A, Immunofluorescence images at 10×; *YAP* ^f/f^;*Tek*‐Cre mice showed delays in the development of blood vessels and astrocytes at P3, and the blood vessels and astrocytes did not reach the edge of the retina at P7. The white arrow represented the distance between the retina equator and the edge of vessels/astrocyte networks (μm), the bar = 100 μm. B, Immunofluorescence images are 100×; P3 and P7 *Tek*‐Cre; *YAP* ^f/f^ mice showed reductions in blood vessel sprouting and astrocyte number, as well as the disappearance of cell junctions and baseboards, bar = 10 μm. C, P3 and P7 astrocyte percentage area, astrocyte process length, number of junctions and number of branch points were analysed by AngioTool, and the P7 distance from the retina equator to the edge of vessels/astrocytes was analysed by Sigma Plot. D, GFAP expression was tested by Western blot and showed decreased expression in *YAP* ^f/w^;*Tek*‐Cre and the *YAP* ^f/f^;*Tek*‐Cre mice compared with *YAP* ^f/f^ mice, at P3, P7; N = 3 in (A‐C) (*P < .05 vs *YAP* ^f/f^ mice); N = 5 (D) (*P < .05 vs *YAP* ^f/f^ mice)

Because YAP plays an essential role in retinal vessel malformation, we examined YAP that affects both vessels and astrocyte development. Whole‐mount images of blood vessels and astrocytes showed a sparse vessel organization and delayed development of the astrocyte network in *YAP* ^f/w^; *Tek*‐Cre and *YAP* ^f/f^;*Tek*‐Cre mice (Figure 2A). *YAP* ^f/w^;*Tek*‐Cre mice also exhibited impaired vessel sprouting from the optic nerve head (ONH) and a reduction in astrocyte processes, and these changes were even more apparent in *YAP* ^f/f^;*Tek*‐Cre mice at P3 and P7 (Figure 2B). AngioTool analysis at P3 and P7 revealed striking reductions in the percentage area, number of junctions, total length and number of branching points in retinal astrocytes of YAP conditional knockout (*YAP* cKO) mice, and we also found that the distance from retina equator to the edge of vessels/astrocyte network was the longest in *YAP* ^f/f^;*Tek*‐Cre mice (Figure 2C). The expression of GFAP decreased significantly in both *YAP* ^f/w^;*Tek*‐Cre and *YAP* ^f/f^;*Tek*‐Cre group compared to *YAP* ^f/f^ group (Figure 2D). Since YAP expression was deleted in ECs, the malformation of astrocytes was thought to occur through a secondary and cell nonautonomous cellular mechanism. GFAP staining was also dismissed in the heterozygous and homozygous *YAP* cKO mice and demonstrated that the typical marker of mature retinal astrocytes was reduced by the vessel defect. In addition, the loss of YAP not only damaged the retina, but also damaged the blood‐brain barrier in mice at early growth (Figure S2).

### 3.3 YAP deficiency of retinal ECs contributes to the genesis of immature astrocytes

Previous studies have shown that mature astrocytes maintain a stable and ordered developmental origin from normal differentiation of immature astrocytes.^23^ Vascularization delay induces abnormal astrocyte proliferation, migration and maturation in the mouse retina,^9^, ^24^, ^25^ and, as demonstrated above, EC YAP deletion alters astrocyte phenotype. Therefore, we speculated that YAP in ECs might regulate the maturation of astrocytes.

**Figure 3:**
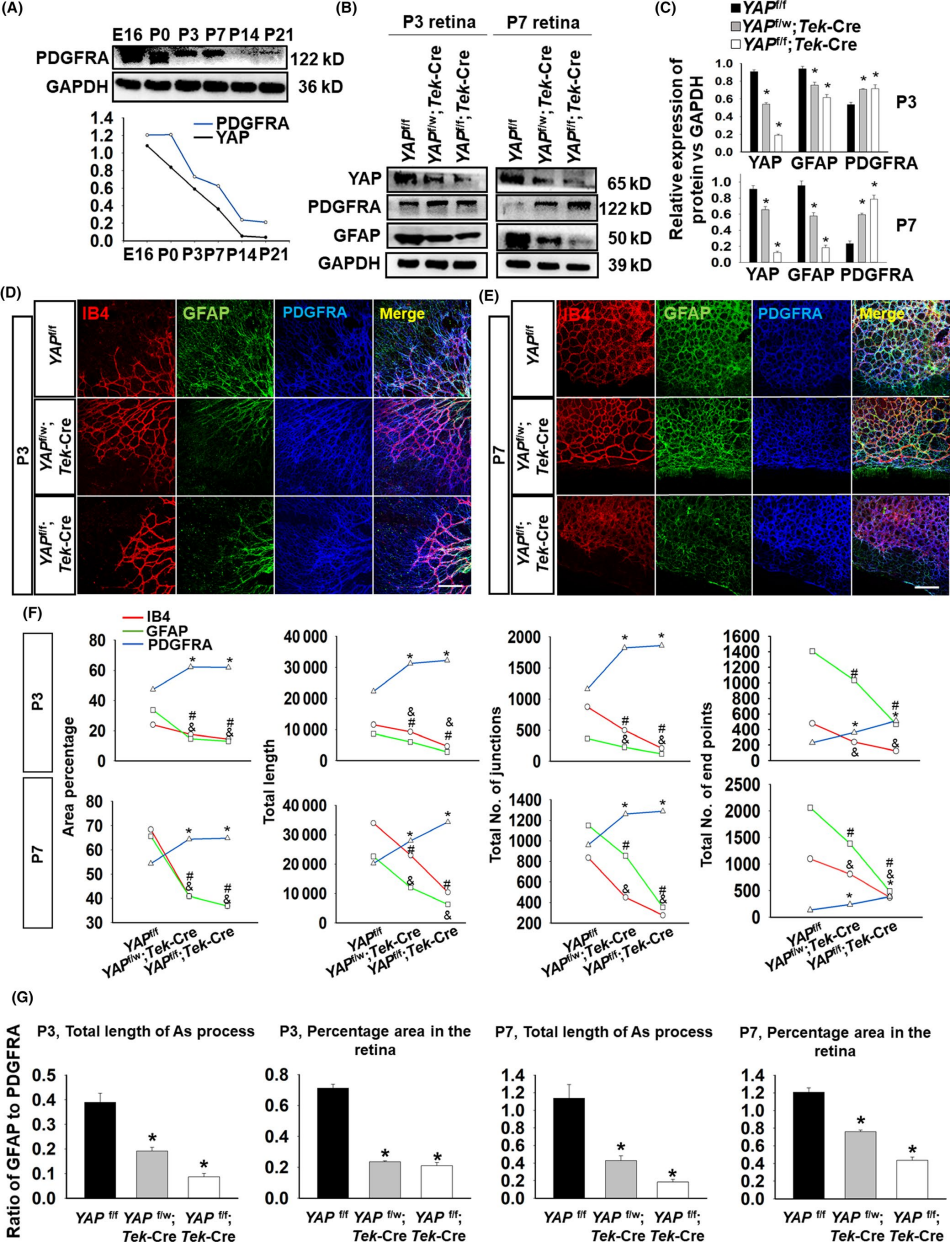
YAP deficiency of retinal ECs contributes to the genesis of immature astrocytes. A, Western blot showed that PDGFRA expression representing immature astrocytes was consistent with YAP expression and declined from E16 to P21 (PDGFRA is shown in blue and YAP in black). B, C, At P3 and P7, *YAP* cKO mice showed a declining trend in YAP and GFAP expression but an increase in PDGFRA expression, especially at P7 (*YAP* ^f/w^ groups are in black, *Tek*‐Cre; *YAP* ^f/w^ in grey, and *Tek*‐Cre; *YAP* ^f/f^ in white, n = 5, *, *P* < .05). D, The growth of blood vessels and astrocytes stained by IB4 and GFAP was slower than the growth of immature astrocytes stained by PDGFRA in cKO mice at P3. E, PDGFRA+ astrocytes reached the edge of the retina when blood vessels and mature astrocytes did not in *Tek*‐Cre; *YAP* ^f/f^ mice at P7, bar = 50 μm. F, P3 and P7 vessel percentage area, total vessel length, total number of junctions and total number of end points were analysed by AngioTool. These results all showed the same declining trend in cKO mice, IB4 in red line, GFAP in green and PDGFRA in blue. G, The ratio of mature (GFAP+ positive signal) and immature (PDGFRA+ positive signal) in the retina were analysed by Sigma Plot. N = 5(B‐C) (*P < .05 vs *YAP* ^f/f^ mice); N = 3 (D) (*p _PDGFRA_ < 0.05 vs *YAP* ^f/f^ mice; ^#^p _GFAP_ < 0.05 vs *YAP* ^f/f^ mice; ^&^p _IB4_ < 0.05 vs *YAP* ^f/f^ mice); N = 3 (G) (*P < .05 vs *YAP* ^f/f^ mice)

Our study found that the expression of immature retinal astrocytes (labelled by platelet‐derived growth factor receptor A, PDGFRA) gradually decreased from E16 to P21 which was consistent with the expression pattern of YAP in retinal development (Figure 3A). Compared to the littermate controls (*YAP* ^f/f^) mice, *YAP* cKO mice showed obviously lower expressions of YAP and GFAP at P3 and P7 but remarkably higher expressions of PDGFRA (Figure 3B,C). Compared to the *YAP* ^f/f^ littermates, *YAP* ^f/w^;*Tek*‐Cre and *YAP* ^f/f^;*Tek*‐Cre mice exhibited a higher PDGFRA fluorescence intensity but lower GFAP fluorescence intensity, indicative of a growing number of immature retinal astrocytes at P3 and P7 (Figure 3D,E). We compared the percentage area, length, junction and end point parameters among *YAP* ^f/f^, *YAP* ^f/w^;*Tek*‐Cre and *YAP* ^f/w^;*Tek*‐Cre littermates by assessing different markers, such as IB4, GFAP and PDGFRA, and analysed these parameters using AngioTool. The significant reduction in IB4 and GFAP expression was accompanied by an increase in PDGFRA expression in *YAP* cKO mice (Figure 3F). Furthermore, we compared the ratio of mature and immature cells in the retina with YAP deleted in single or both alleles (Figure 3G).

These results demonstrated that *YAP* cKO increased the number of immature astrocytes, which may be a crucial cause of the maturation disorder observed in astrocyte development.

### 3.4 EC‐expressed YAP governs LIF secretion to regulate astrocyte maturation

**Figure 4:**
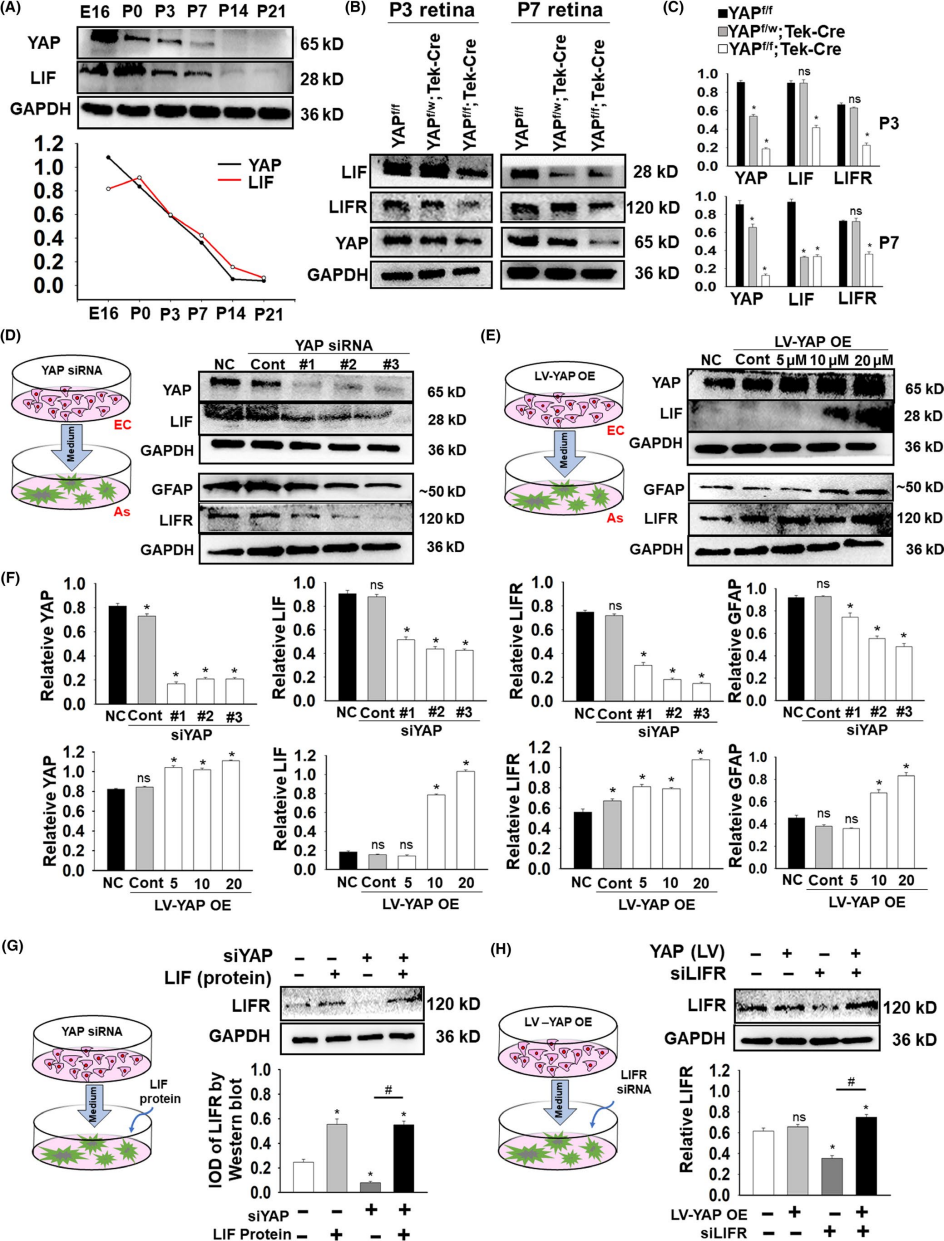
EC‐expressed YAP governs LIF secretion to regulate astrocyte maturation. A, Western blot showed that LIF protein expression decreased from E16 to P21, consistent with YAP expression (LIF is in red and YAP is in black). B, C, At P3 and P7, cKO mice showed a declining trend in LIF expression, which was consistent with that in YAP expression, especially at P7 (*YAP* ^f/w^ groups are in black, *Tek*‐Cre; *YAP* ^f/w^ in grey, and *Tek*‐Cre; *YAP* ^f/f^ in white). D‐F, Model images showed the coculture system, and Western blot results demonstrated that YAP expression in HMEC‐1 cells decreased after YAP siRNA transfection and increased after YAP LV infection. LIF and LIFR expression levels were consistent with YAP expression. G, Model images the process of coculture system, and LIFR expression on cocultured astrocytes was tested in four groups. The Western blot results showed that the lowest LIFR expression levels were in the LIFR siRNA‐only group and that the addition of YAP LV increased LIFR expression. H, The Western blot results showed that the lowest LIFR expression was in the YAP siRNA‐only group, and LIF protein addition promoted LIFR expression regardless of YAP siRNA. N = 5 (B‐C) (*P < .05 vs *YAP* ^f/f^ mice); N = 5 (D‐F) (^ns^p > 0.05; *P < .05 vs NC group); N = 3 (G) (^ns^p > 0.05; *P < .05 vs NC group; ^#^ *P* < .05 comparison between LIFR siRNA and YAP LV infection); N = 3 (H) (^ns^p > 0.05; *P < .05 vs NC group; ^#^ *P* < .05 comparison between YAP siRNA and LIF protein addition)

Leukaemia inhibitory factor is secreted by ECs and combines with LIFR on astrocytes to affect astrocyte maturation.^26^ According to our findings, LIF expression was consistent with YAP expression across postnatal retina development (Figure 4A). In the YAP cKO groups, LIF expression was reduced in early growth (Figure 4B,C). We hypothesized that YAP in retinal ECs affects the secretion of LIF during development.

To verify the relationship between endothelial YAP, LIF and astrocytic LIFR, we knocked down or overexpressed YAP in the HMEC‐1 line. According to the Western blot results, HMEC‐1 YAP knockdown (#1, #2, #3 siRNA) decreased LIF and LIFR expression, and YAP overexpression increased it (Figure 4D‐F). We then reduced YAP expression by siRNA in HMEC‐1 cells and added LIF protein to the coculture medium in one experiment and overexpressed YAP in HMEC‐1 cells by LV and knocked down LIFR expression in astrocytes in another one. LIF protein promoted LIFR expression on astrocytes, which was suppressed by YAP siRNA (Figure 4G). Furthermore, HMEC‐1 YAP overexpression increased LIFR expression on astrocytes, which was inhibited by LIFR siRNA (Figure 4H).

Thus, we deduced that YAP controlled EC LIF secretion to govern LIFR on astrocytes, which is crucial for maturation, in vivo and in vitro.

## 4 DISCUSSION

Astrocytes and blood vessels play important role not only in retinal development but also in retinopathy. Since astrocytes form a reticular network that appears to provide a substrate for EC migration, they have long been proposed to guide angiogenesis.^27^ Despite their important relationship in normal development, the factors that control vascularization and astrocytogenesis of the retina remain underdetermined. In this study, we discovered a new astrocyte‐vascular interaction mechanism by which the deletion of YAP, an essential coactivator in the Hippo signalling pathway, governed retinal astrocytic differentiation and maturation by decreasing LIF secretion in adjacent ECs. We showed that *Tek*‐Cre*;YAP^f/f^* mice provide a robust model for clarifying the molecular events in retinal vasculature formation and related astrocyte remodelling. Furthermore, our data indicated that LIF acts as an organizer in regulating retinal astrocyte maturation.

### 4.1 YAP is required for retinal vessel development and parallel retinal astrocyte maturation

YAP is a highly conserved transcript coactivator of endothelial cells and is known to play an essential role in EC distribution, vascular development and maintenance.^11^, ^28^, ^29^ YAP knockout is lethal due to developmental arrest of the embryo and severe defects in the yolk sac vasculature.^30^ Here, we used an endothelial‐specific *Tek*(Tie2)‐Cre transgenic mouse line to specifically delete YAP in ECs.^31^ Only a fraction of ECs in the aorta and common atrial chamber showed a *Tek*‐driven lacZ‐positive signal at E8.5 and later at E9.5. Most of the ECs were labelled with lacZ, which suggested that the specific *Tek* promotor drove the functional loss of YAP from retinal ECs beginning at E9.5. *Tek*‐Cre mice have been widely used for retinal vascular research.^32^, ^33^, ^34^ Consistent with a previous study,^35^ the ECs in the tip of the vascular front region of *YAP* cKO mice exhibited a blunted end with fewer dysmorphic filopodia (Figure 2B). Since a previous study showed that TAZ cKO mice appeared normal and had no obvious vascular phenotype, the current study examined *YAP* cKO mice, which exhibit a gene dose‐dependent dysfunctional vascular phenotype.^36^ Our studies also revealed gene dose‐dependent effects on the retinal vascular phenotype and resulting astrocytic phenotype in *YAP* homozygous and heterozygous cKO mice (Figure 2A‐C), suggesting that the primary vessel defect contributes to secondary astrocyte malformation.

A quantity of proliferating astrocytes has been shown to accompany developing vessels as they migrate across the primate retina from the optic nerve, playing an important role in vessel stabilization and pathological neovascularization.^37^, ^38^, ^39^ Furthermore, astrocytes provide a gradient of vascular endothelial growth factor A (VEGFA) that directs retinal angiogenesis.^40^ However, recently, the sequential induction model of astrocyte and retinal angiogenesis has been challenged by suggestions that substances secreted from ECs might also change astrocyte characteristics,^9^, ^19^, ^25^ including astrocyte proliferation, differentiation, migration and maturation. Consistent with these observations, we noticed a delayed retinal vessel network‐related astrocyte degeneration in the *YAP* cKO genetic mouse model (Figure 2). In P14 and P21 *YAP* cKO mice, a sparse vascular network was found in superficial, intermediate and deep plexus, which to be affected by YAP deletion (Figure S3A‐E). However, little difference of glial process and density was found in P14 and P21 retina, which suggesting primary autonomous vascularization is not a sufficient cause after retinal astrocyte maturation (Figure S4a‐e). The invading astrocyte process and vessel model are presented in Figure S4f. Furthermore, YAP takes essential role in vascular barrier maturation by arranging the distributions of tight and adherens junction proteins, maintaining the barrier integrity.^34^ Thus, we could not exclude the secondary effect of disrupted retinal barrier integrity in astrocytic undernourishment which needs further study to elucidate. Hence, the network of astrocytes and ECs was destroyed correspondingly, suggesting a prominent role of vessel cytoplasmic YAP in regulating astrocyte sprouting.

### 4.2 YAP governs astrocytic maturation via the secretion of LIF in the endothelium

GFAP is a typical astrocyte marker that represents retinal mature astrocytes and muller cells. GFAP overexpression is a hallmark of reactive gliosis (RG), the major pathophysiological feature of retinal damage. We found an elimination of GFAP staining in the edge of the retina in *YAP* cKO mice accompanying the reduction in endothelial tip cells (Figure 2A‐C). Instead, dense PDGFRA+ astrocytes (immature) were located in parallel with the endothelial tip cells (Figure 3D‐F), which is consistent with a previous study.^25^ Anatomical analysis revealed that low‐level GFAP‐expressing astrocytes first invade the retina, gradually express higher levels of GFAP, and become quiescent.^20^, ^41^ The presence of PDGFRA+ astrocytes in the avascular region was attenuated at P7, which suggests that the maturation of astrocytes in *YAP* cKO mice is partially compensated by other signalling pathways that require further elucidation. Astrocyte maturation is regulated by classic regulators, such as SOX2, which is a highly conserved transcriptional factor in all stages of central nervous system development.^42^ Dentin matrix protein 1‐proteoglycan (DMP1‐PG)^43^ and astrocytic contact^44^ are also required for astrocyte maturation. Mature astrocyte markers also include S100β, Aldh1L1, AldoC, Ascgb1, Glt1 and aquaporin 4.^45^ Nevertheless, GFAP has been widely used as a specific marker to examine dynamic changes in astrocyte maturation.^46^ While compensating for the weak expression of GFAP, PDGFRA from retinal ganglion cells promotes the growth of immature astrocytes expressing it,^40^ making PDGFRA an immature astrocyte marker. The abundant overgrowth of immature astrocytes in the avascular region in *YAP* cKO mice is consistent with the phenotypes of apelin or APJ deficiency.^25^ The decrease in LIF expression with *YAP* deletion suggests that the immaturities of astrocytes might be secondary to the lack of vessel‐originated YAP (Figure 4). Meanwhile, as LIF could be produced by different cell types in the retina such as RGC and glial cell, we would not exclude the possibility of the origin of LIF from other cell types. LIF‐YAP is an axis that governs not only breast cancer metastasis and cell polarity^47^, ^48^ but also embryonic stem cell self‐renewal.^49^ Astrocyte maturation, differentiation and angiogenesis are promoted by introducing LIF^19^, ^20^, ^50^ via different signalling pathways, such as signal transducer and activator of transcription‐3 (STAT3).^51^ However, YAP‐LIF signalling has not yet been well established between vessels and related astrocytes. In the current study, we observed a correlation between the downregulation of YAP and LIF *Lif* KO mice have been reported to exhibit overall hypervascularity in their retinas, consistent with our finding that *YAP* cKO mice showed hypervascularity by restricting migration to the front of the vascular network area at P7 rather than P3, unlike *APJ* KO mice.^25^ Alternatively, *YAP* cKO induced hypoxia due to insufficient outgrowth of blood vessels, which may then induce hypervascularity caused by LIF reduction (in ECs) and VEGF upregulation (in immature astrocytes). Although EC‐released LIF has been demonstrated to induce astrocyte maturation,^19^, ^20^, ^25^ how LIF secretion is induced in ECs and the upstream mechanism has not been determined. LIF secretion was estimated under the control of oestrogen receptor (ER) during embryo implantation.^52^ Previous studies reported G protein‐coupled oestrogen receptor (GPER) stimulation activates YAP and transcriptional coactivator with a PDZ‐binding domain (TAZ), via the Galphaq‐11, PLCβ/PKC, and Rho/ROCK signalling pathways.^53^ Moreover, Integrin‐FAK signalling is a novel IL‐6 and LIF regulation mechanism relevant to the inflammation and stem cell fields,^54^ and also contributes to stem cell‐based tissue renewal regulation^55^ and tumour budding of colorectal cancer^56^ in YAP signalling. Nevertheless, ER or integrin‐YAP‐LIF axis still needs further detailed elucidation. Herein, the current data revealed that EC‐derived YAP is an upstream regulator of LIF and governs astrocyte maturation, which plays essential roles in regional retinal angiogenesis. Deletion of YAP in ECs not only regulates EC proliferation, migration and survival^57^ but also abrogates LIF‐related astrocyte maturation and hypervascularity.

## CONFLICT OF INTEREST

The authors declared that there is no conflict of interest.

## AUTHOR CONTRIBUTION

ALQY, YRD and CX performed most of the experiments on retina whole‐mount staining, Western blotting, immunostaining and cell transfection. LYJ, LWY, ZJY, ZZ and CCL conducted imaging experiments and data analysis. LS initiated the research, designed research studies, analysed data, provide the funding and wrote the manuscript. Yan J. and Ye J. conducted the research, analysed data and provide the funding. All authors approved the final version of the manuscript.

## Data Availability

The authors declare that all data supporting the conclusions of this study are presented within the paper and the supplementary information files and are available from the authors.
