# Supplementary figures and images for: Retinal blood vessel‐origin yes‐associated protein (YAP) governs astrocytic maturation via leukaemia inhibitory factor (LIF)
Source: Cell Prolif. 2020 Jan 8;53(2):e12757. doi: 10.1111/cpr.12757 (PMC7046482; doi:10.1111/cpr.12757)

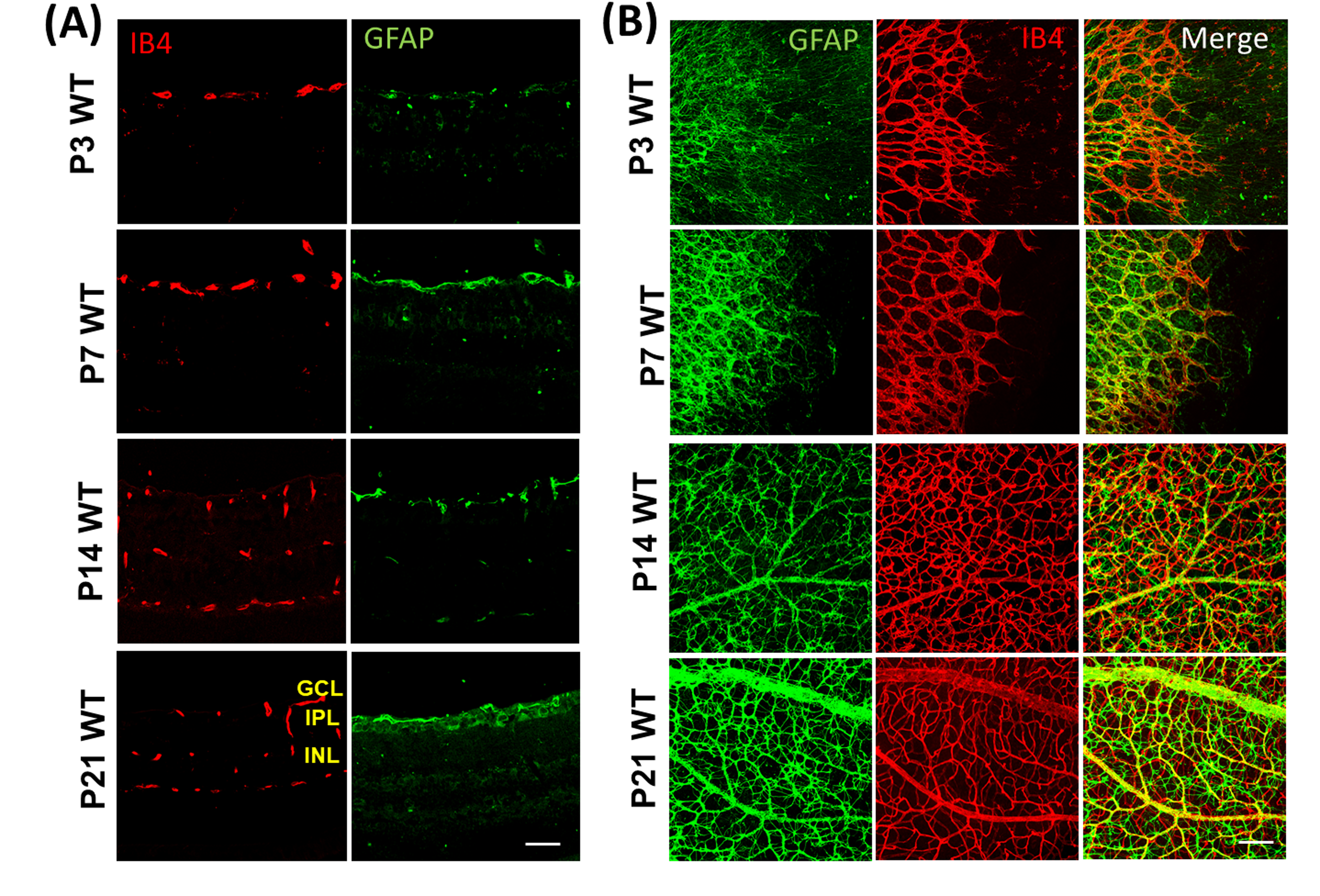

Supplement: Supplementary file 1 [file CPR-53-e12757-s001.tif]

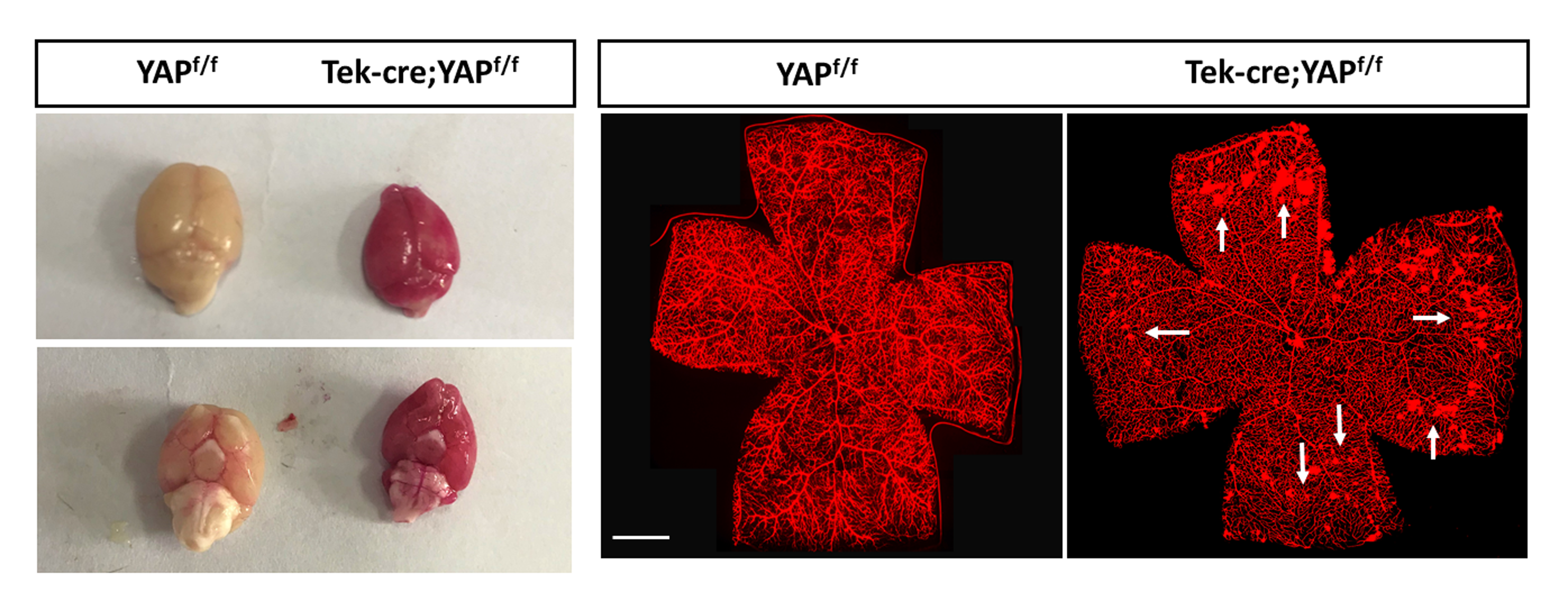

Supplement: Supplementary file 2 [file CPR-53-e12757-s002.tif]

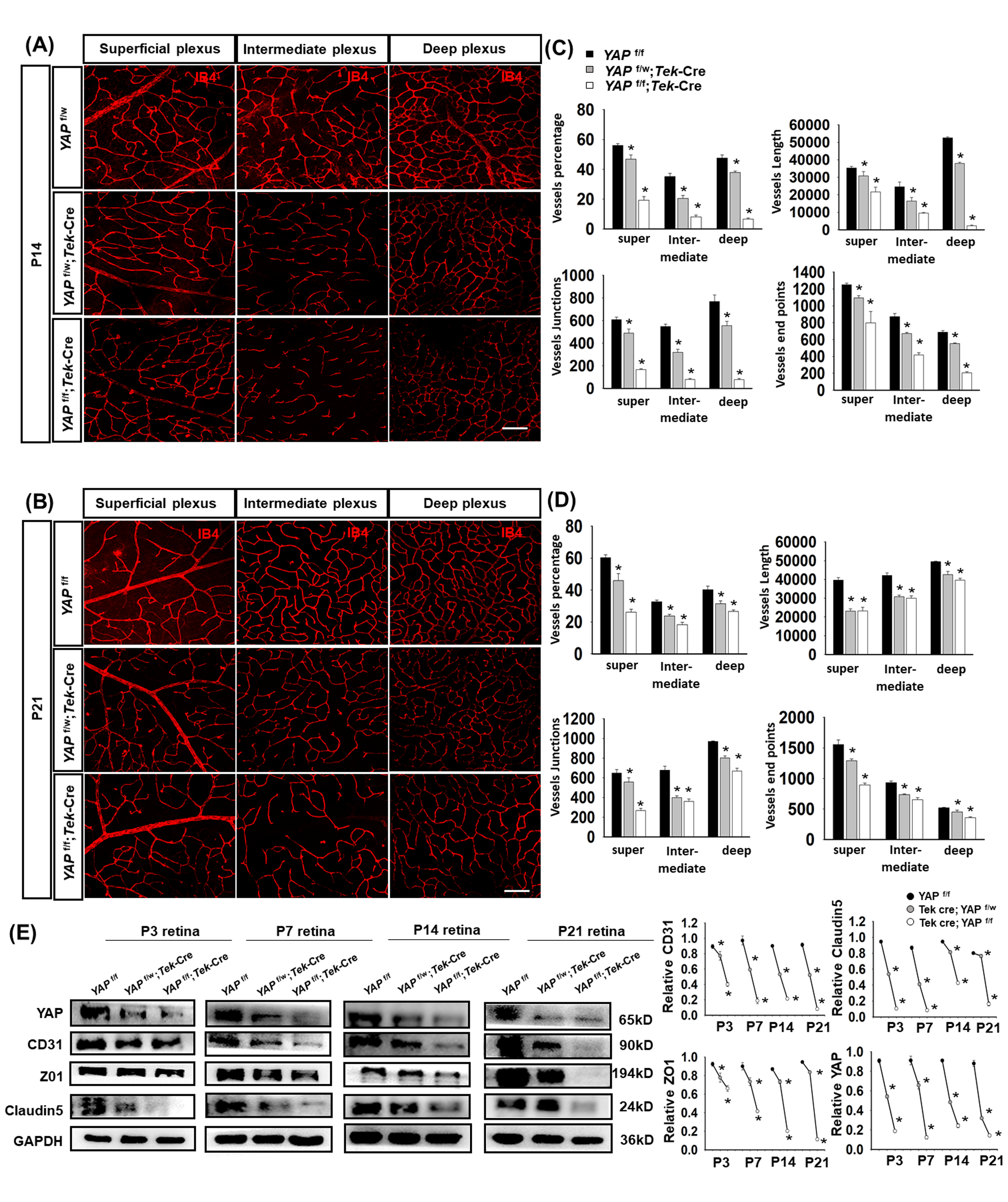

Supplement: Supplementary file 3 [file CPR-53-e12757-s003.tif]

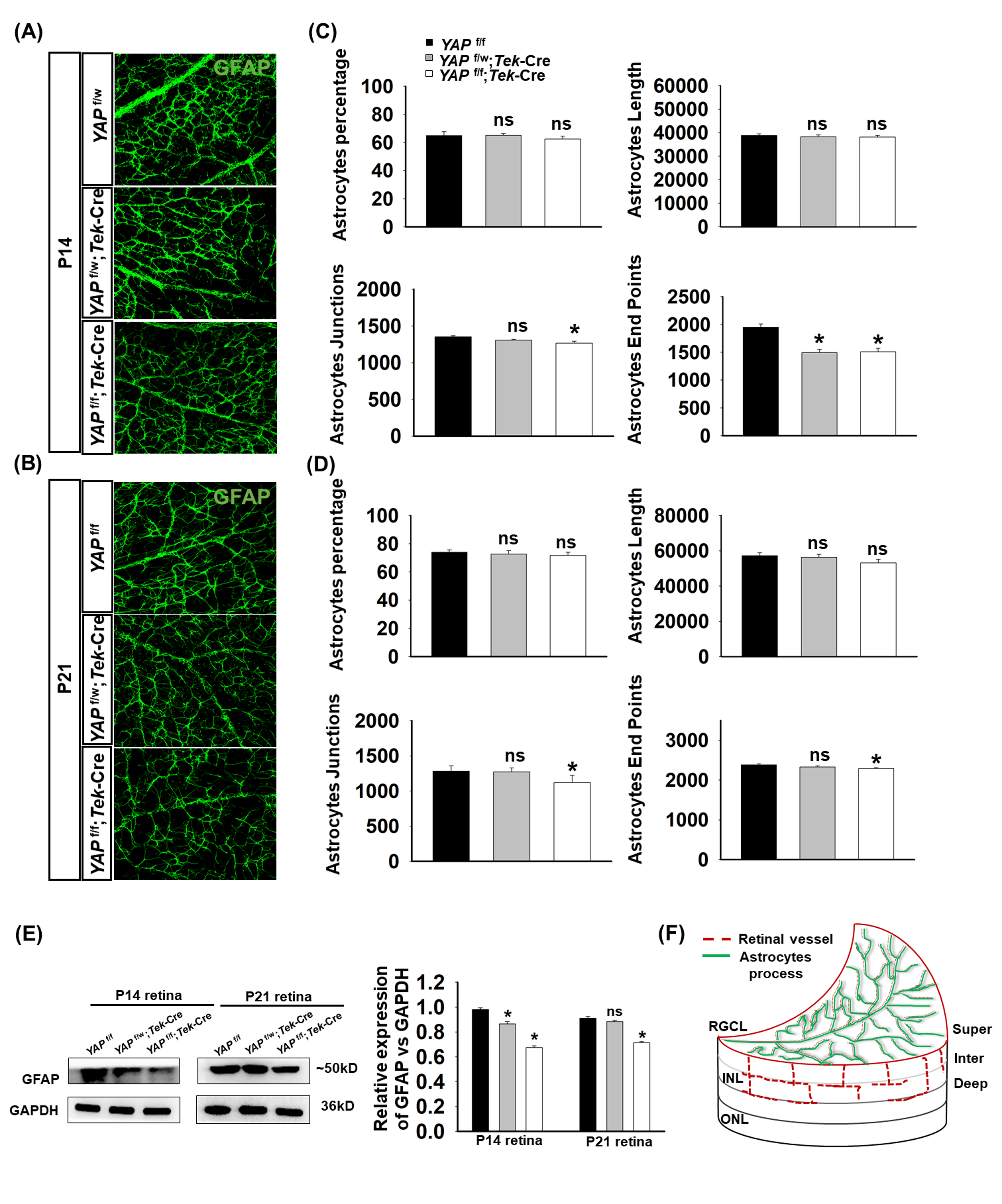

Supplement: Supplementary file 4 [file CPR-53-e12757-s004.tif]
